# Ureteral metastasis from pulmonary adenocarcinoma: A case report and literature review

**DOI:** 10.1111/1759-7714.14172

**Published:** 2021-10-20

**Authors:** Wang Shen, Jindong Chen, Limin Gao, Guangzhi Ma, Lan Yang, Hao Liang, Jingsi Dong, Qinghua Zhou

**Affiliations:** ^1^ Lung Cancer Center West China Hospital, Sichuan University Chengdu China; ^2^ Intensive Care Unit West China Hospital, Sichuan University Chengdu China; ^3^ Department of Pathology West China Hospital, Sichuan University Chengdu China; ^4^ Department of Respiratory and Critical Care Medicine West China Hospital, Sichuan University Chengdu China

**Keywords:** pulmonary adenocarcinoma, targeted therapy, ureteral metastasis

## Abstract

The occurrence of ureteral metastasis from distant primary tumors is uncommon, and appears to be especially rare when it originates from the lungs. In the case presented here, a patient with lumbago and left hydronephrosis was diagnosed with left ureteral metastasis of pulmonary adenocarcinoma after a CT‐guided percutaneous transthoracic needle biopsy of the lung and retroperitoneal laparoscopic left nephroureterectomy. He accepted the targeted therapy because the lung tumor epidermal growth factor receptor mutation (exon19 deletion) was positive, and preoperative staging of lung adenocarcinoma was stage IVA. After an 8‐month follow‐up, he is still alive and well, with no local recurrence or distant metastases. The therapy outcome assessment is stable disease. Although rare, our case has demonstrated that pulmonary adenocarcinoma has the possibility of metastasizing to the ureter, a risk that should be considered in some lung cancer patients.

## Graphical abstract

A patient with lumbago and left hydronephrosis was diagnosed with left ureteral metastasis of pulmonary adenocarcinoma after a CT‐guided percutaneous transthoracic needle biopsy of the lung and retroperitoneal laparoscopic left nephroureterectomy. Although rare, our case has demonstrated that pulmonary adenocarcinoma has the possibility of metastasizing to the ureter, a risk that should be considered in some lung cancer patients.

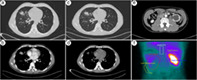

## INTRODUCTION

Lung cancer continues to be the main cause of cancer‐related deaths in both men and women worldwide.^1^ About 57% of lung cancer cases are discovered at an advanced stage when the cancer has spread to other organs.^2^ The brain, bones, liver, and adrenal glands are all common metastatic sites. Ureteral metastasis, on the other hand, is quite uncommon. According to a review of the literature, Cohen et al. discovered only 31 cases of metastatic cancer of the ureter in a collection of nearly 3200 corpses, with breast, colon, and lymphoma being the most prominent primary sources of metastatic ureteral lesions.^3^ The report by Fitch et al. showed that these lesions, which included the breast, stomach, bladder, colon, cervix, rectum, prostate, ovary, and melanoma‐skin, eye, comprised more than 80% of the primary sites of metastatic ureteral tumors, with the lung accounting for only 2.5% of metastatic ureteral tumors.^4^ This case study features a 37‐year‐old lung cancer patient with left ureteral metastases.

## CASE REPORT

**FIGURE 1:**
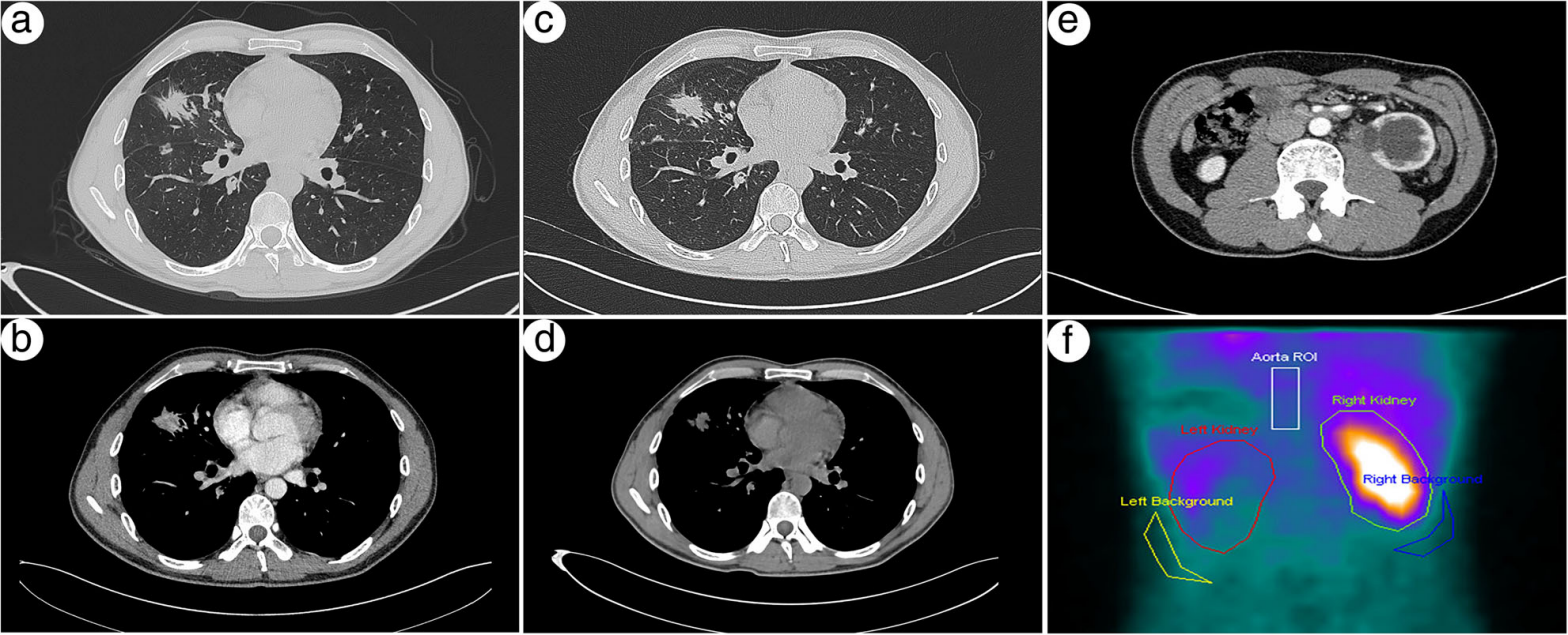
(a,b) Before targeted therapy, chest computed tomography (CT) images revealed a 3.0 cm × 2.4 cm irregular soft tissue mass with heterogeneous enhancement located in the lateral segment of the right middle lobe. (c,d) After 8‐month targeted therapy, chest CT images showed a 2.6 cm × 2.3 cm irregular soft tissue mass. The tumor volume decreased when compared to prior treatment. (e) Abdominal CT images revealed enlargement of the left kidney, a soft tissue nodule with heterogeneous enhancement at the proximal end of the ureter, dilation and hydronephrosis of the left renal pelvis and calyces, thinning of the renal parenchyma, hydronephrosis of the left kidney, and an unclear boundary between the lesion and the left psoas major muscle. (f) SPECT renal dynamic imaging revealed that the perfusion of the left kidney decreased, the shape blurred, and the right kidney was normal

**FIGURE 2:**
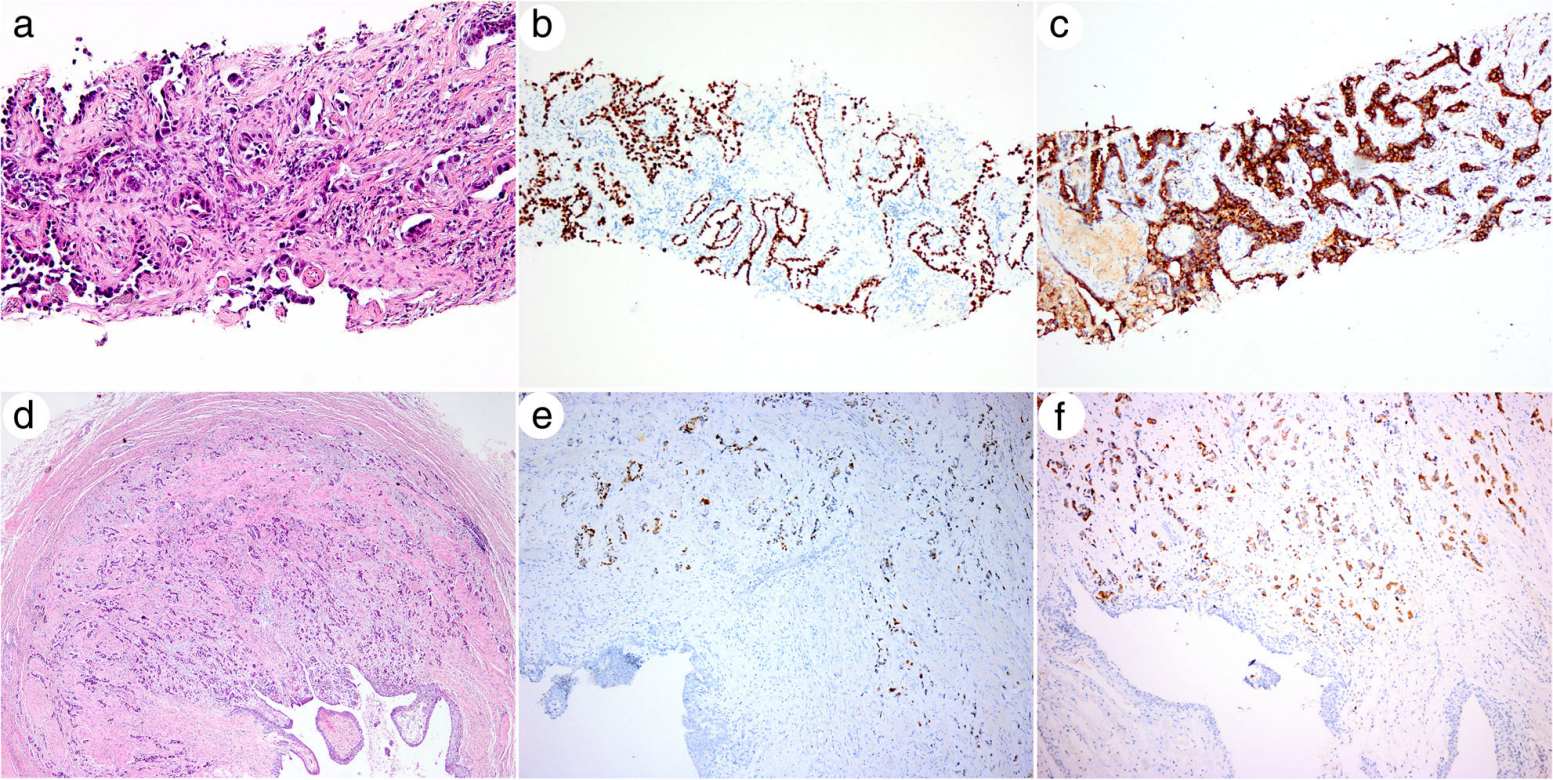
Hematoxylin‐eosin staining and immunohistochemical staining of the primary pulmonary adenocarcinoma (a–c) and the ureteral metastatic adenocarcinoma (d–f). (a) The primary lung adenocarcinoma. (×200). (b) TTF‐1 expression was positive in the nucleus. (×100). (c) Napsin A expression was positive in the cytoplasm (×100). (d) The tumor involved the entire layer of the ureteral wall. (×40). (e) TTF‐1 expression was positive in the nucleus (×100). (f) Napsin A expression was positive in the cytoplasm (×100)

A 37‐year‐old Chinese male presented to the West China Hospital with the main complaint of lumbago of 2‐months duration. He had no fever, cough, dyspnea, hemoptysis, hematuria, abdominal pain, or other respiratory and urinary system symptoms and did not smoke, nor had been exposed to secondhand smoke. No lymphadenopathy or palpable masses were found in the neck. Glomerular filtration rate (GFR) was 38.20 ml/min/1.73 m^2^ (normal, 56–122 ml/min/1.73 m^2^), serum creatinine was 202 mol/l (normal, 68–108 mol/l), carcinoembryonic antigen (CEA) was 116 ng/ml (normal, 5 ng/ml), and circulating tumor cell folate acid receptor (FR‐CTC) was 12.8FU/3 ml (normal, 8.7FU/3 ml), and the remaining laboratory tests, including urine tests, were normal. An enhanced chest computed tomography (CT) scan revealed a 3.0 cm × 2.4 cm irregular soft tissue mass with heterogeneous enhancement in the lateral segment of the right middle lobe, and both bilateral hilar and mediastinal lymph nodes were enlarged (Figure 1a,b). A CT‐guided percutaneous transthoracic needle biopsy of the lung was then performed. The tumor was identified as an adenocarcinoma after pathological examination of lung puncture tissue (Figure 2a). In immunohistochemistry, TTF‐1 (Figure 2b), Napsin A (Figure 2c), CK7, and TTF‐1 (SPT2) were found to be positive, but CDX2 and ALK‐V (D5F3) were negative. A primary adenocarcinoma of the right middle lobe was diagnosed pathologically. An enhanced abdominal CT examination revealed enlargement of the left kidney, a soft tissue nodule at the proximal end of the ureter with heterogeneous enhancement, and dilation and hydronephrosis of the left renal pelvis and calyces (Figure 1e). Single photon emission computed tomography (SPECT) renal dynamic imaging showed perfusion of the left kidney had diminished, the shape was blurred, but the right kidney was normal. The left and right kidneys had GFRs of 14.5 and 44.5 ml/min, respectively (Figure 1f). After excluding the possibility of ureteric calculi, the radiological signs were suggestive of a ureteral tumor. Both brain magnetic resonance imaging (MRI) and whole‐body bone scans revealed no evidence of distant metastases.

On day 7 after admission, the patient underwent a retroperitoneal laparoscopic left nephroureterectomy to ease his urinary obstruction and obtain a pathological diagnosis. Intraoperatively, a grayish white mass, closely attached to surrounding tissue, was detected at the left proximal ureter and renal hilar. On gross pathology, the renal pelvis and calyces were markedly dilated, and the mucosa was smooth. There were no stones, and the difference between the cortex and the medulla was ill‐defined, with the cortex being noticeably thinner. The ureter was approximately 6 cm in length, with a rough mucosal area of about 3.5 cm × 0.7 cm at 2.5 cm from the end. The wall of the ureter was around 0.3–0.4 cm thick, and the lumen had narrowed. The tumor was tough, with a grayish white section. Microscopic observation revealed the tumor was a poorly differentiated adenocarcinoma that involved the entire layer of the wall of the ureter (Figure 2d). Immunohistochemical staining was positive for TTF‐1 (Figure 2e), Napsin A (Figure 2f), PCK and CK7, while GATA‐3, P63 and PAX8 were negative. The final pathological diagnosis was left ureteral metastasis of pulmonary adenocarcinoma, stage IVA. Following that, utilizing the patient's lung puncture tissue, next generation sequencing (NGS) was performed to detect lung cancer‐related driver gene mutations, with the results indicating that *EGFR* mutation (exon19 deletion) was positive. We next used NGS to look for driver gene mutations in the patient's ureteral metastatic tumor tissue, but found none. He was discharged from the hospital after 7 days of hospitalization after a successful postoperative course.

Based on the driver gene mutation and the preoperative staging of lung adenocarcinoma, we had a multidisciplinary team (MDT) meeting to carefully assess the patient's illness and then set a treatment goal appropriate for that stage. Subsequently, he accepted the targeted therapy of osimertinib (a third‐generation EGFR tyrosine kinase inhibitor, TKI). After 8 months of targeted therapy, chest CT showed a 2.6 cm × 2.3 cm irregular soft tissue mass was located in the lateral segment of the right middle lobe. When compared to prior treatment, bilateral hilar and mediastinal lymph nodes remained stable, and the tumor volume had decreased (Figure 1c,d). The therapy outcome assessment is SD. The patient is still alive and well after an 8‐month follow‐up, with no local recurrence or distant metastases.

## DISCUSSION

Lung cancer has a high tendency to metastasis, which is a major predictor of poor prognosis. The brain is the most common metastatic site, followed by the bones, liver, and adrenal glands.^5^ However, metastasis to the eyelids, prostate, and ovaries is infrequently reported.^6^, ^7^, ^8^ Furthermore, ureteral metastases were found in only 2 (0.16%) of the 1281 lung cancer patients evaluated by Babaian et al.,^9^ indicating that ureteral metastases from pulmonary tumors are extremely rare.

Clinically, the presence of ureteral metastases in some lung cancer patients may go unreported until abnormal imaging or renal function tests reveal them. William et al. found only three of the 31 patients with metastatic cancer to the ureter were found to have hematuria, and the majority of cases (85%) were asymptomatic and discovered by chance at autopsy.^3^ According to research by Presman and Ehrlich, some patients with ureteral metastases experienced abdominal pain, lumbago, urine infection, and ureteral obstruction, which were symptoms similar to urolithiasis. Additionally, urine results, which are not specific, may not be able to predict the presence or absence of metastasis‐related secondary ureter involvement.^10^ In a nutshell, misdiagnosis and underdiagnosis are quite possible. As a result, a positron‐emission tomography (PET)‐CT scan or imaging evaluation of the whole abdomen, chest, and head is necessary, which could have a significant impact on diagnosing, staging, and finding local metastases.

Recent progress in molecular biology has resulted in a substantial shift in treatment strategies for non‐small cell lung cancer (NSCLC), from the empirical use of cytotoxic drugs to targeted regimens. In addition, as knowledge has become more specialized, MDT for the management of cancer patients has been developed, consisting of surgeons, oncologists, radiologists, pathologists, and expert nurses.^11^ In our case, according to radiologists, radiation therapy raises the risk of local edema, which can lead to urine retention and post renal failure. On the other hand, we believe that the optimal surgical options can completely remove ureteral lesions and provide a clear pathological diagnosis. This mode, as seen in our case, can benefit patients by multiperspective analysis and optimizing treatment plans.

To summarize, regular and thorough re‐examinations of lung cancer patients are required and should be carried out to detect metastases early. Despite its rarity, urinary system metastases should be suspected when a lung cancer patient exhibits signs of impaired renal function, ureteral obstruction, or hydronephrosis. In individuals with advanced NSCLC who have distant solitary metastasis, surgery can relieve symptoms while also providing a precise pathology diagnosis that can aid treatment. Furthermore, the implementation of an MDT therapy plan can optimize treatment and be beneficial for patients.

## CONFLICT OF INTEREST

No authors report any conflict of interest in this work.
